# Mechanism and treatment of olfactory dysfunction caused by coronavirus disease 2019

**DOI:** 10.1186/s12967-023-04719-x

**Published:** 2023-11-17

**Authors:** Bian Hu, Mengdan Gong, Yizhen Xiang, Siyuan Qu, Hai Zhu, Dong Ye

**Affiliations:** 1 Department of Otorhinolaryngology-Head and Neck Surgery, The Affiliated Lihuili Hospital of Ningbo University, Ningbo, 315040 Zhejiang China; 2 Department of Otorhinolaryngology-Head and Neck Surgery, Ninghai First Hospital, Ningbo, 315600 Zhejiang China

**Keywords:** Olfaction, Olfactory dysfunction, COVID-19, SARS-CoV-2

## Abstract

Coronavirus disease 2019 (COVID-19) is an infectious disease caused by the severe acute respiratory syndrome coronavirus 2 (SARS-CoV-2). Since the start of the pandemic, olfactory dysfunction (OD) has been reported as a common symptom of COVID-19. In some asymptomatic carriers, OD is often the first and even the only symptom. At the same time, persistent OD is also a long-term sequela seen after COVID-19 that can have a serious impact on the quality of life of patients. However, the pathogenesis of post-COVID-19 OD is still unclear, and there is no specific treatment for its patients. The aim of this paper was to review the research on OD caused by SARS-CoV-2 infection and to summarize the mechanism of action, the pathogenesis, and current treatments.

## Introduction

The spread of severe acute respiratory syndrome coronavirus 2 (SARS-CoV-2) has had negative impacts on public health and economic development worldwide. With the start of the global pandemic, it was reported that many Coronavirus disease 2019 (COVID-19) patients experience olfactory dysfunction (OD) during the course of their disease and/or after recovery [1, 2]. Olfaction plays a key role in social communication, personal safety, and diet enjoyment. As such, abnormal olfactory function can have varying degrees of adverse effects on psychological and cognitive functions and can reduce the quality of life of affected individuals.

An epidemiological survey showed that 63–78% of patients with post-COVID-19 OD experience complete or partial restoration of olfactory function within 30 days after onset [3]. However, some reports suggest that patients can experience symptoms of OD for 1–2 years or even longer after COVID-19 [4, 5]. Currently, it is widely recognized that COVID-19 causes OD, but the exact pathogenesis is not yet clear. Therefore, this paper summarizes the epidemiological characteristics, pathogenesis, and treatment of post-COVID-19 OD and provides some reference basis for the rational management of patients and related clinical practice.

## Methods

A literature review of PubMed and Google Scholar was conducted to find studies related to olfactory dysfunction in the context of COVID-19. Search terms included: olfactory dysfunction, anosmia, olfaction, smell, SARS-CoV-2, COVID-19, treatment, and olfactory training, and there were no time restrictions for search. Studies were then screened by two different readers to ensure they met the inclusion benchmarks. Relevant information was also screened from the reference lists of selected articles. Articles containing misleading titles were excluded. Lack of clarity of methodology and research with weak study design were excluded from the review. Included studies had to discuss olfactory dysfunction in human or animal models of COVID-19, and further explore the pathogenesis and treatment of OD in COVID-19.

## The epidemiology of OD following COVID-19

In an early 2020 study of 41 patients with COVID-19 in Wuhan, researchers found that these patients often presented with clinical symptoms such as fever, cough, muscle pain, and fatigue and less frequently with hemoptysis, diarrhea, and dyspnoea [6]. Meanwhile, in another study of 99 patients with COVID-19 pneumonia in China, documented clinical manifestations included fever (83%), cough (82%), muscle aches (11%), shortness of breath (31%), confusion (9%), headache (8%), runny nose (4%), sore throat (5%), chest pain (2%), nausea and vomiting (1%), and diarrhea (2%), while olfactory symptoms of hyposmia were not present [7]. However, since March 2020, there has been a significant worldwide increase in reports of OD due to COVID-19. In the United Kingdom [8], the incidence of OD displayed an exponential increase similar to the rise in COVID-19 cases during February and March 2020. Some academics determined that more than half of homebound patients with COVID-19 and their household contacts as well as hospitalized patients in the London area had abnormal olfactory function, and they suggested that OD could be used as an evaluation criterion in programs to identify cases and guide case isolation [9, 10].

Previously, it was determined that the incidence of OD in COVID-19 patients among 31 provinces of Iran was highly correlated with the incidence of COVID-19 during the same period [11]. An Italian study comparing the incidence of OD in COVID-19 patients isolated at home and hospital inpatients showed that the incidence of OD was lower among inpatients than among those isolated at home [12]. This is consistent with the findings of Ben-Chetrit et al. who found that OD was a common symptom in home-isolated COVID-19 patients and their household contacts, with an incidence of 63.0% [13]. Another case–control study revealed that COVID-19 patients were more likely to have olfactory and gustatory disturbances than influenza patients and that OD was usually the first symptom of COVID-19 [14]. Similar findings have been reported in Asian case cohorts, where it has been suggested that patients infected with SARS-CoV-2 have a greater incidence of olfactory and gustatory disturbances than those with other respiratory viral infections [15]. This suggests that OD is not only one of the common manifestations of COVID-19 but also likely to be the first or only symptom experienced by COVID-19 patients [16]. Therefore, in the context of a major worldwide SARS-CoV-2 outbreak, it is important for clinical staff to be more alert to patients with sudden-onset OD.

## Mechanism of OD caused by COVID-19

Many viruses can cause nasal mucosal edema, thereby preventing odor from entering the olfactory cleft and binding to olfactory receptors, temporarily affecting the patient’s odor perception [17]. Studies have shown that inflammatory edema can occur in the bilateral olfactory clefts of COVID-19 patients. Therefore, nasal obstruction is considered to be a possible mechanism of OD caused by COVID-19 in the early stage [18, 19]. However, a number of studies have reported that patients with common viral rhinitis often recover their sense of smell after the nasal obstruction is relieved, while nearly 60% of COVID-19 patients continue to experience OD after nasal patency [20]. This indicates that olfactory cleft obstruction is not the main mechanism of OD in patients with COVID-19. In addition, the incidence of OD was higher in COVID-19 patients (31.65%) than in influenza patients (10%) [21]. The above results suggest that the pathogenesis of OD in COVID-19 patients is somewhat specific. It has been suggested that the mechanism of action may be as follows: SARS-CoV-2 is induced by angiotensin-converting enzyme 2 (ACE2) and transmembrane serine protease 2 (TMPRSS2), which are expressed by non-neuronal cells in the olfactory epithelium (OE) [22–25]; SARS-CoV-2 directly invades the OE and causes damage to olfactory neurons [26–29]; and SARS-CoV-2 causes a cytokine storm that can cause damage to the nervous system, including olfactory receptors [30–33].

### SARS-CoV-2 invades the olfactory system

The OE is the peripheral organ of the olfactory system and exists in the mucosa of the olfactory fissure. It covers the upper part of the nasal septum, the roof, the superior turbinate, and the anterolateral part of the middle turbinate. The OE is mainly composed of olfactory sensory neurons (OSNs) and non-neuronal cells such as olfactory sustentacular cells (OSCs), basal cells, Bowman's gland cells, and microvillar cells [30]. Odor metabolizing enzymes are present in the OE, and uridine diphosphate glucuronosyltransferase (UGT) is a major odor metabolizing enzyme. UGT membrane proteins are categorized into two major groups UGT1 and UGT2, the latter of which includes UGT2A and UGT2B. They play an important role in peripheral olfactory processes by catalyzing the rapid biotransformation of odorants leading to their elimination or synthesis of new odor stimuli [34–36]. Recent research reported a genome-wide significant locus in the vicinity of the UGT2A1 and UGT2A2 genes. This study showed that compared with non-infected people, the people infected with SARS-CoV-2 are 11% more likely to lose their sense of smell due to genetic variation at its locus [37]. Polymorphisms in the UGT2A1/UGT2A2 locus are associated with an increased risk of acute olfactory loss associated with COVID-19, and the gene product is expressed in OSCs, consistent with the primary location of infection [33, 37]. These genes may play a role in the physiology of infected cells that contribute to the loss of olfactory capacity.

Studies have shown that the entry of SARS-CoV-2 into cells depends upon the expression of both ACE2 and TMPRSS2 in target cells [22]. It has been demonstrated that SARS-CoV-2 infects cells via the interaction of its spike protein with ACE2 and on target cells, which requires cleavage and excitation of the spike protein by the cellular protease TMPRSS2 [23]. Hendawy et al. also found by gene sequencing that two essential genes (*ACE2* and *TMPRSS2*) associated with SARS-CoV-2 entry into cells were expressed in both the mouse and human olfactory mucosa [24]. Immunohistochemical studies have additionally suggested that the ACE2 protein is widely expressed in the dorsal OE of mice in periorbital cells and supporting cells. Bryche et al. used a golden Syrian hamster model to study for the first time the effects of SARS-CoV-2 infection in the nasal cavity; these authors employed confocal double-labeled immunostaining to determine which cells were infected with SARS-CoV-2 in OE, then found that OSCs were rapidly infected with SARS-CoV-2 only 2 days after nasal perfusion. This infection is related to the recruitment of a large number of immune cells in the lamina propria, followed by rapid degradation of OE [25]. This observation is consistent with the expression patterns of ACE2 and TMPRSS2 in these cells. Transmembrane protein 16F (TMEM16F) is a Ca^2+^-activated chloride channel and scramblase, which can be expressed in OE cells, especially in supporting cells [38]. Recent studies have shown that TMEM16A and TMEM16F can be activated by SARS-CoV-2, leading to elevated Cl^−^ secretion [39], and the spiking proteins on the host cell surface interact with ACE2 receptors on neighboring cells to promote the fusion process by anchoring neighboring cells [40–42]. Supporting cells can simultaneously express ACE2 and TMEM16F, therefore, when SARS-CoV-2 infects the OE, intercellular interactions form a wide range of syncytia, which affects olfactory function.

**Fig. 1:**
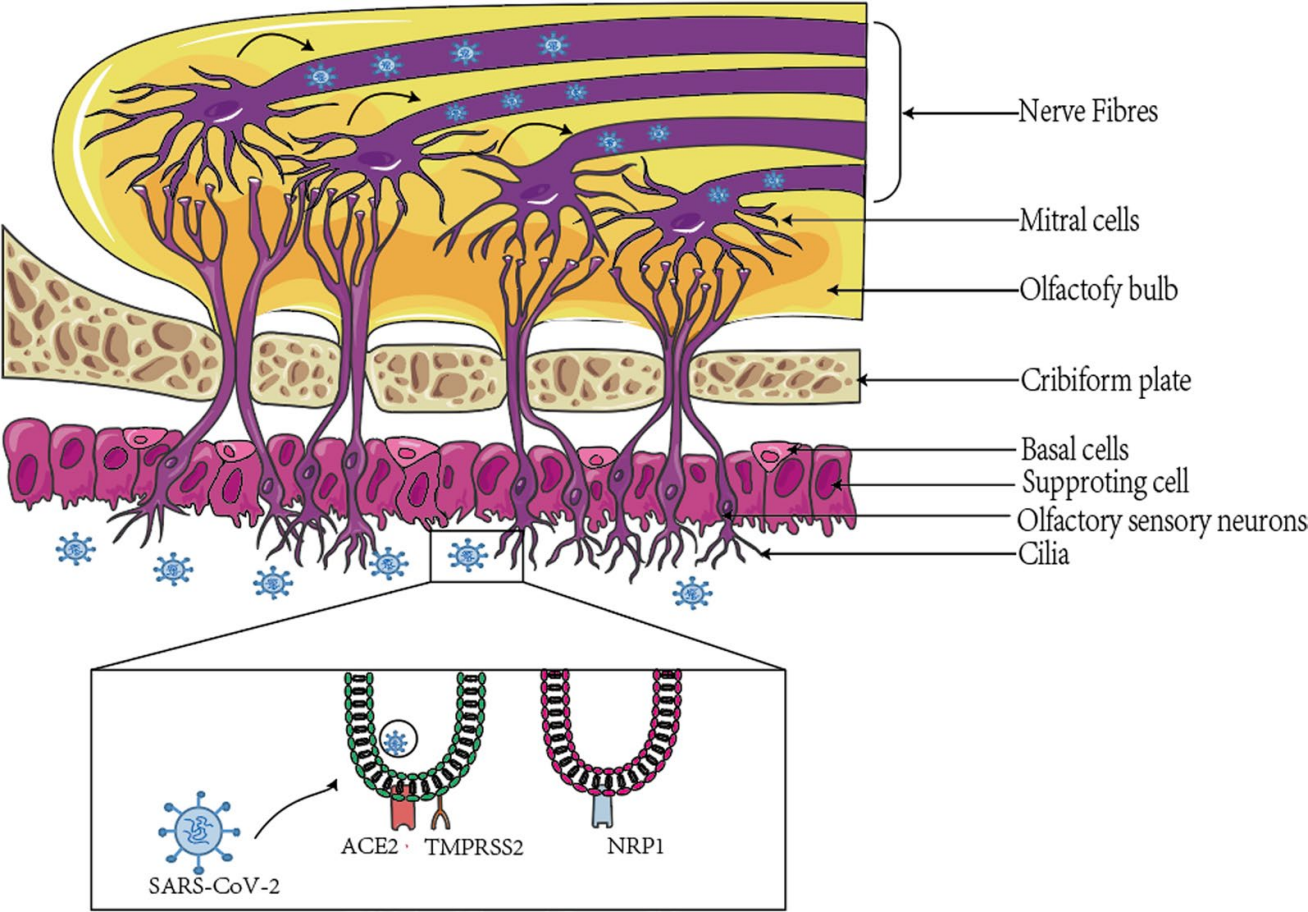
Severe acute respiratory syndrome coronavirus 2 invades the olfactory epithelium

In addition, some studies have found a bypass pathway mediated by the neuropilin-1 receptor (NRP1) for SARS-CoV-2 entry, which can also bind with the virus’ spike protein and promote viral entry into cells. Studies have found that almost all olfactory cells (including OSNs) can express a large amount of NRP1 [43–45]. Cantuti et al. performed an autopsy study of patients who died from complications of COVID-19 and found that NRP1 was highly expressed in infected human olfactory epithelial cells [26]. Therefore, the OE cells with high expression of these proteins are believed to be the main target of SARS-CoV-2. The mechanism of SARS-CoV-2 invasion into the OE is shown in Fig. 1.

In the central nervous system, the olfactory bulb (OB) is an important hub in the olfactory neural circuit. Analysis of imaging revealed that the OB volume of COVID-19 patients with OD was significantly lower than that of normal controls [46], and some patients had structural abnormalities of the OB (e.g., left–right asymmetry [47], changes in morphology [48]) or signal abnormalities [48]. Studies based on viral RNA and protein detection have found SARS-CoV-2 infections in the OB of COVID-19 patients [4, 49], viral antigens were detected in the outer layer of the OB [50, 51]. Researchers speculated that the structural changes of the OB in patients are mainly derived from the damage of the virus to the supporting cells and stem cells of OE [52]. SARS-CoV-2 caused massive destruction of the OE cells and axonal damage to olfactory nerve fibers [53], which in turn led to the absence of trophic factors in the OB [54]. The damage of stem cells prevents normal regeneration of the olfactory epithelium, resulting in structural changes in the image of the OB a few weeks after infection [55].In addition, neuroimaging studies have suggested a reduction in the volume of the orbitofrontal cortex in COVID-19 patients [56] and a decrease in metabolic activity [47]. Alterations in the orbitofrontal cortex, which receives secondary olfactory projections and is associated with olfactory awareness, may also contribute to the olfactory deficits caused by SARS-CoV-2.

**Fig. 2:**
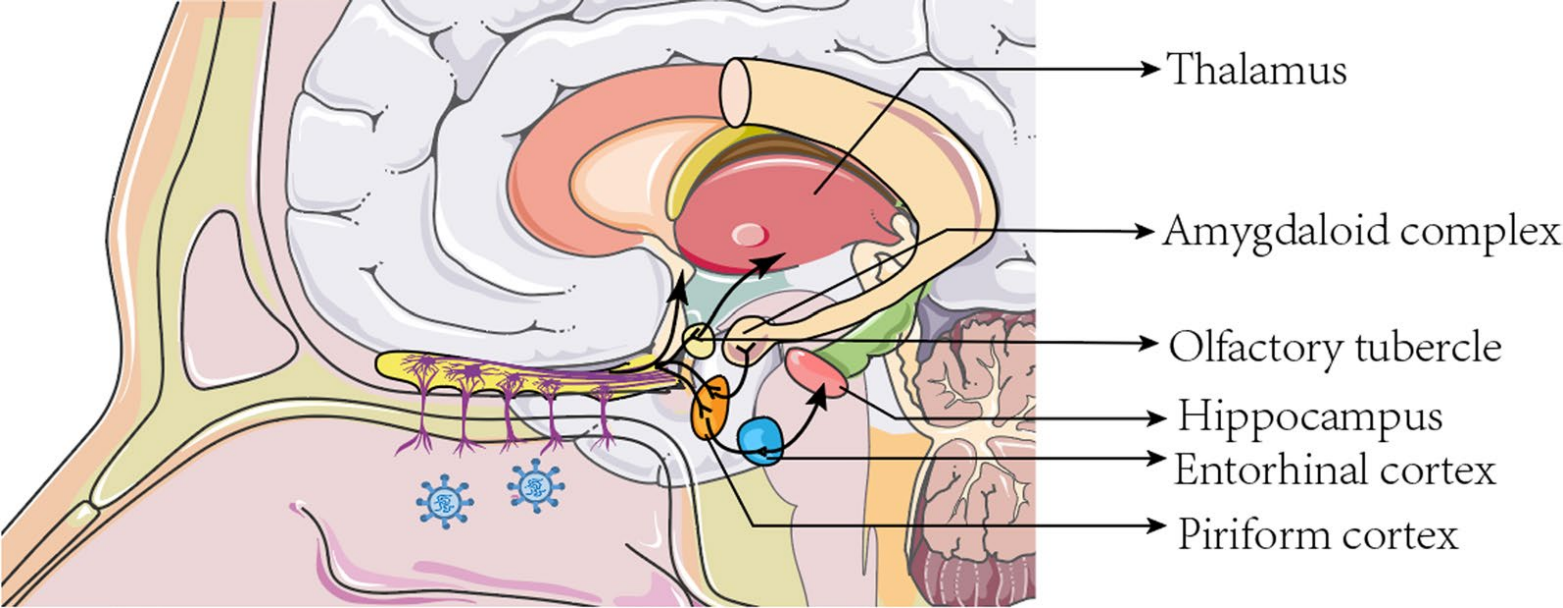
Diffusion of severe acute respiratory syndrome coronavirus 2 in the brain

The specific pathways and mechanisms by which SARS-CoV-2 affects the central nervous system are controversial. SARS-CoV-2 may also damage the central olfactory system through retrograde neural pathways or blood sources, causing OD. The basal cells of olfactory receptors are located in the OE. After the OE is infected, the virus may spread to the basal cells and then to the mature olfactory neurons [57]. These infected olfactory neurons are synaptically linked to the OB, which is linked in turn to the central nervous system, allowing for viral transmission to the brain, where it can spread rapidly, representing a potential pathway for central nervous system infection [49, 58] (see Fig. 2). In addition, SARS-CoV-2 can cross the blood–brain barrier via the circulation and enter the central nervous system. It was demonstrated that perivascular cells of the OB highly express the ACE2 protein, which is essential for maintaining the blood–brain barrier and mediating neuroimmune responses. Thus, infection of these cells can alter OB blood perfusion or induce an inflammatory response that indirectly affects the function of olfactory neural pathways. Stoyanov et al. further confirmed that histopathological changes in the OB and frontal lobes of the brain of COVID-19 patients may be associated with their olfactory impairment and that their OB tissue exhibited necrotizing olfactory bulbitis [59]. In addition, Butowt et al. proposed another hypothesis that SARS-CoV-2 may be transmitted directly from olfactory epithelial non-neuronal cells to the cerebrospinal fluid surrounding the olfactory nerve bundle near the sieve plate before spreading later to most areas of the brain, including the medulla oblongata, which is the respiratory and circulatory center in the brainstem [27].

### Damage to OSNs

**Fig. 3:**
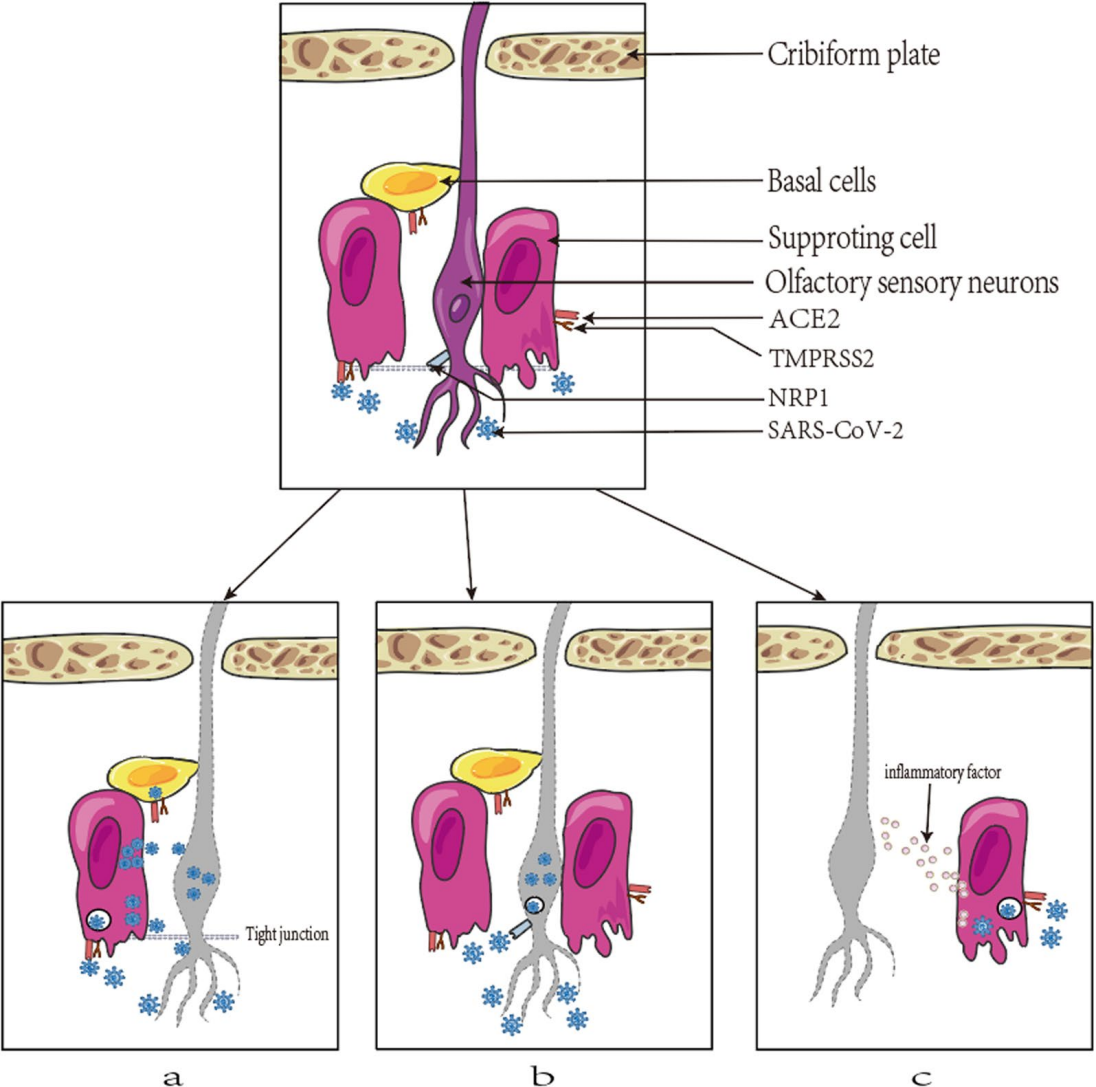
Severe acute respiratory syndrome coronavirus 2 (SARS-CoV-2) damages olfactory sensory neurons (OSNs). **a** Supporting cells affect OSN functions through tight junctions. **b** SARS-CoV-2 enters and damages OSNs. **c** Amplification of inflammatory factors leads to OSN impairment

The odor receptors on the dendritic cilia of OSNs in the OE first detect odors. The olfactory nerve axons pass through the skull base through the sieve plate and are connected to the OB to become mature OSNs, transmitting olfactory information to the advanced center. Some studies have shown that abnormalities in olfactory function and apoptosis of neuronal cells are closely linked. Although OSNs do not express ACE2 receptors, SARS-CoV-2 was still found to be present in mature OSNs in a hamster model of viral infection [57], a finding which may be related to the high expression of NRP1 in OSNs, which mediating direct access and damage to OSNs by SARS-CoV-2 [26]. In contrast, supporting cells express high levels of ACE2 and TMPRSS2. OSCs, which are considered to be partly glial and partly epithelial in nature, respectively, are the supporting cells of OE and the key to proper odor perception. Studies have shown that OSCs can wrap a large fraction of the OSN dendrites in OE and provide them with neurotrophic signaling and physical support. They also act as phagocytes to remove dead OSNs [60, 61]. SARS-CoV-2 first infects OSCs and then OSNs through the tight connection between them, inhibiting the olfactory conduction cascade [27]. Therefore, damage to the OSCs can indirectly destroy the function of OSNs. In addition, COVID-19 may also trigger an immune and inflammatory response, leading to neuronal cell death and olfactory impairment. It has been reported that the inflammatory response may affect the normal structure and function of olfactory neurons by inducing apoptosis and death programs, decreasing the number of olfactory neurons and thus affecting the normal functioning of olfaction [28, 29], as shown in Fig. 3.

### Immune response affects the olfactory system

When the body is exposed to pathogens or otherwise severely stimulated, the immune system is over-activated and a rapid, massive release of multiple cytokines in body fluids creates a cytokine storm. Excessive levels of inflammatory markers such as interleukin (IL)-6 and tumor necrosis factor (TNF)-α, which are caused by cytokine storms, can damage olfactory neurons [30, 31]. Viral immunology studies have shown that SARS-CoV-2 can bind to ACE2 in the body, leading to high expression levels of IL-1β, IL-6, and TNF-α, triggering a cytokine storm that leads to multi-organ damage in the body. In addition, it has been found that immune cells, such as macrophages, can infiltrate into the OE soon after infection [32]. Deep transcriptional profiling of olfactory epithelial cell types by flow cytometry revealed that microvilli cells (MVCs) and a small number of OSNs may be involved in the inflammatory response to SARS-CoV-2 infection. Infected cells, such as supporting cells, can form syncytia with cells that do not express ACE2, compromising nearby OSNs and interfering with their function while also initiating a rapid immune response in MVCs and a fraction of OSNs, leading to the migration of activated lymphocytes toward the OE and inducing the production of pro-inflammatory cytokines [31]. In addition, it has been demonstrated after the elimination of SARA-CoV-2 from the tissue for a long time, there was still a response to persistent inflammatory signals in the olfactory epithelial cells, and the number of OSNs also decreased, suggesting a mechanism of long-term OD after COVID-19 [33].

### Neurotransmitter abnormalities causing OD

SARS-CoV-2 infection may affect the normal function of neurons, including the function of components of the olfactory nervous system, through a variety of pathways. Among these, interference with neurotransmitter release may be a more critical factor with a causative role in the onset of olfaction abnormalities.

It has been suggested that SARS-CoV-2 may affect neurotransmitter release by infecting the brain and olfactory nerve cells. The key to entry of SARS-CoV-2 into host cells is its binding to ACE2 and TMPRSS2 on the cell surface; since the cell surfaces in both the cerebral nervous system and olfactory nerves contain ACE2 and TMPRSS2 receptors, it is expected that SARS-CoV-2 will infect these cells in this way [62]. In addition, related studies have reported that SARS-CoV-2 may also use other receptors on the cell surface to directly invade the cerebral nervous system and olfactory nerve cells, but the specific receptors and mechanism of action are not yet clear [63, 64]. The mechanism of interference with neurotransmitter release after SARS-CoV-2 infection also needs to be further investigated, and it is speculated that it may be related to the induction of apoptotic programming by SARS-CoV-2 in neuronal cells through the induction of neuronal death. It is also possible that SARS-CoV-2 infection may induce an immune response in the body, promoting the release of inflammatory cytokines, which causes damage to neuronal cells, leading to neuronal cell death and ultimately affecting neurotransmitter release [65]. It has been reported that SARS-CoV-2 can inhibit vesicle transport on cell membranes and reduce intracellular neurotransmitter release [66]. The mechanism of action in this context may be as follows: impeding the fusion of vesicles and cell membranes, which in turn prevents the release of neurotransmitters from vesicles; activating excessive immune responses in the body, destroying intracellular microtubules and vesicle structures; and interfering with the structure and stability of microtubules, thus preventing the normal localization of vesicles and neurotransmitter transport and inhibiting neurotransmitter releas.

### Vascular damage affects olfactory function

It has been shown that invasion by SARS-CoV-2 may cause vascular damage, which in turn leads to a lack of oxygen and nutrient supply to olfactory cells, triggering OD [67].

The entry of SARS-CoV-2 into vascular endothelial cells is the first step in the onset of vascular injury. SARS-CoV-2 virus binds to ACE2 receptors on the surface of host cells through the surface spinosin, which is an important step in its entry into the host cell. SARS-CoV-2 can be mediated by ACE2 receptors to enter vascular endothelial cells. During ACE2 receptor–mediated entry, the SARS-CoV-2 stinger protein needs to be sheared by TMPRSS2 to facilitate its entry into the host cell [68]. In contrast, TMPRSS2 is also expressed in vascular endothelial cells, implying that SARS-CoV-2 may enter vascular endothelial cells via ACE2- and TMPRSS2-mediated entry. Studies have shown that, in addition to ACE2- and TMPRSS2-mediated entry into the endothelium, SARS-CoV-2 may also enter vascular endothelial cells by other means, such as direct membrane fusion or binding to other cell surface proteins [23, 69]. Once SARS-CoV-2 enters the endothelium, it begins to replicate itself, causing cell damage or even death, resulting in damage to the vessel wall. This renders the blood vessels more susceptible to inflammation. SARS-CoV-2 infection can also cause blood-clotting disorders, which can lead to microvascular thrombosis and obstruction of blood flow, culminating in hypoxic necrosis of local tissues [70]. The interaction of these multiple factors further leads to vascular endothelial cell damage and apoptosis, thereby increasing the extent of vessel wall damage. It has also been reported in the literature that SARS-CoV-2 infection may also provoke a cytokine storm, causing an inflammatory response and vascular injury [71, 72].

In summary, SARS-CoV-2 infection may cause vascular damage, resulting in impaired blood transport in the nasal cavity and reduced blood flow to the epithelial cells of the nasal mucosa and olfactory nerve cells, which in turn may affect the normal function of olfaction and result in olfactory impairment.

### Persistent OD in patients with “Long COVID”

“Long COVID” generally refers to the symptoms that persist for more than 3 months after infection with SARS-CoV-2 [4]. A meta-analysis showed that 90% of patients recovered their olfactory function within 90 days after SARS-CoV-2 infection, but 5% still had persistent OD after half a year [73]. There are relatively few empirical studies exploring the mechanisms of persistent OD in patients with “Long COVID”. Overall, SARS-CoV-2 may cause long-term damage to the olfactory system through both direct invasion of OE cells and induction of an inflammatory response. There may also be complex interactions between the two approaches.

SARS-CoV-2 can directly invade body cells through ACE2 and TMPRSS2 receptors. Both basal cells and supporting cells can express ACE2 and TMPRSS2 receptors, which are conducive to the direct invasion of SARS-CoV-2, and these two cells play a vital role in the regeneration and function maintenance of OSNs. It has been hypothesized that persistent infection of olfactory epithelial stem cells (e.g., horizontal basal cells) by SARS-CoV-2 may cause a long-term decline in olfactory epithelial regenerative capacity [74–76]. Another hypothesis suggests that persistent SARS-CoV-2 infection can also cause apoptosis in a large number of olfactory-related cells (e.g., supporting cells). Under normal circumstances, supporting cells can regenerate from the stem cells of the olfactory epithelium, but persistent infection slows down the rate of regeneration, and the recovery of olfactory function will be delayed, resulting in persistent OD [77].

It has been demonstrated that SARS-CoV-2 can affect olfactory function in patients with “Long COVID” by inducing a chronic inflammatory response that simultaneously destroys OSNs and inhibits their regeneration. The up-regulated expression of inflammatory cytokine IL-6 was found in the biopsy samples of the olfactory mucosa of patients with “Long COVID” [78]. Analysis of OE samples from patients with persistent OD showed that long-term T cell-mediated chronic inflammation still existed after virus clearance, and the number of mature OSNs decreased significantly [79, 80]. Therefore, the chronic inflammatory response caused by SARS-CoV-2 can cause continuous immune attacks on OSNs, and the number of OSNs continues to decrease, resulting in persistent OD. Other researchers have proposed that the inflammatory environment and the continued production of inflammatory cytokines (e.g., TNF-α) may also reduce the differentiation potential of horizontal basal cells, inhibit their differentiation to form OSNs, and result in impaired regeneration of OSNs, leading to the persistence of OD [74]. In addition, SARS-CoV-2 was able to persist in the OB of patients even after they had recovered from the acute infection, which resulted in persistent OD [78].

## Treatment of OD in COVID-19 patients

### Drug treatments

#### Corticosteroids

The most common treatment for OD, especially after upper respiratory tract infection, is topical and oral corticosteroids, which are effective in around 25–50% of cases, while oral and nebulized treatments are more effective, and nasal spray administration is less effective, respectively [81]. There remains a lack of scientific basis and conclusive evidence as to whether topical nasal corticosteroids can be given for the treatment of olfactory impairment due to SARS-CoV-2 infection, although it is recommended to continue using nasal corticosteroids rather than discontinuing them in allergic rhinitis patients infected with SARS-CoV-2 [82]. Recent studies suggest that, although nasal corticosteroids do not prevent olfactory impairment due to SARS-CoV-2 infection, they may play a role in reducing the severity and duration of olfactory impairment [83, 84]. A double-blind, randomized, multi-center clinical study showed that nasal irrigation combined with systemic corticosteroids significantly improved olfactory function in patients with COVID-19 with persistent OD lasting for more than 30 days [85]. Some scholars have also found that it is difficult to determine whether drugs such as corticosteroids are therapeutically effective for OD since subjective symptoms disappear after 1 month in most patients who experience a loss of smell after SARS-CoV-2 infection [86, 87]. Therefore, the use of nasal or systemic corticosteroids is also not recommended for patients with post-COVID-19 OD. Meanwhile, the British Rhinological Society (BRS) Consensus Guidelines suggested that oral corticosteroids therapy is recommended for the treatment of OD as an isolated symptom for more than 2 weeks or after other COVID-19 symptoms have resolved, nasal corticosteroids are recommended for patients with OD for more than 2 weeks with nasal symptoms [88].

#### Antioxidant

Oxidative stress, which results from a disruption of the balance between reactive oxygen species and protective antioxidants, plays an important pathogenic role in a variety of diseases, including viral infections. Alpha-lipoic acid (ALA) possesses potent antioxidant and neuroprotective properties and has been shown to reduce serum inflammatory cytokine levels and inflammation-related symptoms in patients with acute coronary syndromes, liver transplants, etc [89]. Hummel et al. found in a prospective uncontrolled clinical trial that 61% of patients with postviral OD had a modest improvement in their olfaction after treatment with ALA [90]. Dragomanova et al. considered that the pharmacological properties of ALA make it a potential candidate drug for the treatment of SARS-CoV-2 infection [91]. However, there is still a lack of research on the use of ALA in the treatment of post-COVID-19 OD, so it is not recommended in the BRS consensus for COVID-19-related OD patients with a condition of more than 2 weeks [88].

Retinoic acid (RA) is a metabolite of vitamin A, and its signaling pathway plays an important role in the embryonic development of the olfactory system and the regeneration of mature neurons [92]. Studies suggested that RA may improve olfaction by promoting OE regeneration, and its modulation of immune function contributes to cellular maintenance, clearance, and transit in the olfactory pathway [93]. Sousa et al. found that RA as an adjuvant therapy can improve the olfactory threshold of post-COVID-19 OD patients more significantly [94].

Omega-3 fatty acids have antioxidant and neuroprotective properties that may help reduce severity in patients with COVID-19. A recent prospective non-blind controlled trial demonstrated that the odor threshold of post-COVID-19 OD patients treated with omega-3 fatty acids was significantly improved [95]. The BRS consensus pointed out that patients can choose to increase the intake of omega-3 fatty acids in their diet or supplements after the post-COVID-19 OD exceeds 2 weeks [88].

Palmitoylethanolamide (PEA), an endogenous fatty acid amide, and Luteolin, a natural antioxidant flavonoid, combine in a pharmaceutical dosage form known as CoUltraPEALut, which has capacities of anti-inflammatory, anti-aging, neuroprotective, and neuroregenerative [96]. A recent study found that the CoUltraPEALut can improve persistent OD after “Long COVID” [97]. A meta-analysis showed that CoUltraPEALut was superior to olfactory training (OT) alone for olfactory recovery when used in combination with OT [98].

#### Zinc

As an important immune trace element, zinc can affect the immune response and infection in several ways [99]. Zinc can reduce the entry of SARS-CoV-2 into cells by reducing the expression of ACE-2, inhibiting the fusion with the host cell membrane, and inhibiting the viral RNA-dependent RNA-polymerase [100]. Jiang et al. showed that zinc deficiency can lead to the loss and apoptosis of olfactory ensheathing cells in the OB, while olfactory ensheathing cell deficiency may lead to OD [101]. One study found that patients treated with zinc for COVID-19-associated OD had a significantly lower olfactory recovery time than those who did not receive zinc [102]. This suggests that zinc may have a significant role in shortening the recovery time of patients’ olfaction.

#### Intranasal insulin

Insulin has been found to have a direct role in the alteration of olfactory signaling. The OB is rich in central insulin receptors, so central insulin is converted in the OB [103]. Increased central insulin resistance is associated with a number of diseases that cause OD [104]. Intranasal insulin has been reported to be effective in the treatment of postinfectious OD, where it has no side effects, does not raise blood glucose levels, and also produces better olfactory sensitivity [105]. Mohamad et al. used an intranasal Insulin fast-dissolving film to treat patients with post-COVID-19 OD and found a significant increase in olfactory recognition values and olfactory detection scores [106], suggesting that intranasal insulin can be used to treat patients with post-COVID-19 OD.

### Olfactory training (OT)

OT is a treatment by which patients can improve their olfactory function by inhaling different types of olfactory agents. Recent studies have shown that OT can improve the olfactory function of healthy people of different ages and patients with olfactory disorders caused by various reasons. The mechanism may be related to promoting olfactory nerve regeneration, inducing functional reorganization or structural changes in the brain, and increasing the OB volume [107]. Initial OT involves exposing the patient to four odors: clove, citronella, eucalyptus, and phenylethyl alcohol. The treatment cycle for OT is typically 12 weeks, during which patients sniff and inhale the aforementioned four strong odors twice a day for about 10 seconds [108]. Prolonging the training time and replacing the olfactory agents can also improve the treatment efficiency of OT [109–111].

A study by Kim et al. found that OT for people with post-infection anosmia can improve their ability to distinguish, recognize, and perceive odors [112]. Karamali et al. suggested that OT may also significantly improve the loss of smell caused by trauma or respiratory infection [113]. It was demonstrated that, by modifying the olfactory agent and extending the training time, treatment outcomes could be improved and the success rate of OT was increased [114, 115]. The BRS consensus recommends OT for all patients with persistent OD for more than 2 weeks after COVID-19 infection [88]. Hwang et al. investigated the efficacy of OT for the treatment of post-COVID-19 OD by meta-analysis and found that OT improved olfactory scores in patients with acute or chronic OD. The rate of OD also decreased significantly [116]. Lechien et al. conducted OT for patients diagnosed with COVID-19 in Europe from March to June 2020 until they fully recovered their sense of smell, indicating that OT also has a positive impact on medium- and long-term recovery. During the follow-up of 1 and a half years after infection, it was found that the higher objective olfactory test scores of patients were significantly correlated with the persistence of OT [117]. Thus, OT is beneficial for patients with acute or chronic dysfunction. Early intervention can reduce the disease duration and improve the quality of life of patients. However, training is also helpful after the acute disease phase. Therefore, even if the diagnosis of OD is delayed, OT may have a sufficient effect.

### Nasal saline irrigation

Nasal saline irrigation is a traditional method for respiratory or nasal care. During nasal saline rinsing, saline is used to rinse away dust particles, allergens, and air pollutants from the nasal cavity, which improves mucosal ciliary oscillation, reduces mucosal edema, promotes local blood circulation, and enhances mucosal clearance. The presence of a saline wash also enhances the hydration of the mucosal mucus layer; increases the frequency of ciliary oscillation; and reduces the production of local inflammatory mediators, which is particularly useful for improving muco-ciliary dysfunction and mucus stagnation caused by viral infection [118]. Both hypertonic saline and isotonic saline can reduce inflammation of the nasal mucosa and promote the repair of damaged nasal muco-ciliary epithelium. Several clinical studies have shown that nasal saline irrigation can effectively improve various symptoms of rhinitis and sinusitis caused by bacteria or viruses, including OD [118–122]. Another meta-study showed that nasal saline irrigation can effectively prevent and reduce viral infections. In particular, nasal saline irrigation can effectively improve the nasal symptoms in patients infected with the Omicron strain of SARS-CoV-2 because of its short clinical incubation period (compared to that of the Delta variant), mild clinical symptoms [123](i.e., more than half of patients experience mild or no symptoms, and symptoms mainly occur in the upper respiratory tract and rarely involve the lungs), and high infectivity (3–5 times that than the Delta variant).

### Other therapies

Tissue engineering, stem cell therapy, and gene therapy hold promise as potential treatments for post-COVID-19 OD [75]. Mesenchymal stem cells (MSCs) can alleviate immune dysregulation by secreting anti-inflammatory cytokines and expressing immunomodulatory surface proteins. Intranasal transplantation of MSCs could help to minimize OE damage and loss of olfactory nerve function [124]. Supporting cells in the OE express keratin-18. Researchers have injected keratin-18-binding peptides or antibody-labeled MSCs into the OE of patients, which can maintain the retention rate of MSCs, reduce the inflammatory environment of the OE of patients, and accelerate the recovery of olfactory function [74].

In gene therapy, Sajid et al. found that small interfering RNAs (siRNAs) can attack highly conserved regions of SARS-CoV-2 RNA, so intranasal siRNA formulations could be a therapeutic option for post-COVID-19 OD [125]. Platelet-rich plasma is a platelet concentrate obtained by centrifugation of autologous whole blood, which contains a large number of growth factors and proteins, and some researchers injected it into the olfactory fissure of COVID-19 patients, resulting in a significant improvement in their olfactory discrimination after 3 months [126].

In addition, methods of neuromodulation are under research. Mature OSNs were found to express high levels of dopamine D2 receptor (DRD2), which acts as an inhibitory G-protein-coupled receptor that inhibits the signaling pathway by which odor molecules bind to the receptor. Olfaction was enhanced when local dopamine synthesis in the mouse olfactory mucosa was pharmacologically inhibited, thus confirming that DRD2 in the nasal cavity can serve as a potential peripheral target for olfactory modulation [127].

## Summary and outlook

Patients with COVID-19 experience a high incidence rate of OD, which is evident early in the disease course of mild and moderate cases with significant age and sex differences. It has been shown that OD is one of the clinical symptoms observed in patients with COVID-19, and it may also be the first symptom or even the only symptom in some patients. As such, the importance of OD in the early warning and diagnosis of COVID-19 cannot be overstated.

At present, there is limited research on OD caused by COVID-19. The timing of their onset and their impact on early diagnosis, treatment, and prognosis as well as their exact pathogenesis also remain to be clarified. Although there are several methods based on OT or drugs to treat patients with post-COVID-19 OD, the overall efficiency is not yet satisfactory, which may be related to the fact that the mechanism of OD occurs differently in different patients, and that better targeting of future treatments is needed, pointing the way for our future research.

## Data Availability

Not applicable.

## Abbreviations

COVID-19: Coronavirus disease 2019
SARS-CoV-2: Severe acute respiratory syndrome coronavirus 2
OD: Olfactory dysfunction
ACE2: Angiotensin-converting enzyme 2
TMPRSS2: Transmembrane serine protease 2
OE: Olfactory epithelium
OSNs: Olfactory sensory neurons
OSCs: Olfactory sustentacular cells
UGT: Uridine diphosphate glucuronosyltransferase
TMEM16F: Transmembrane protein 16F
NRP1: Neuropilin-1 receptor
OB: Olfactory bulb
IL: Interleukin
TNF: Tumor necrosis factor
MVCs: Microvilli cells
BRS: British Rhinological Society
ALA: Alpha-lipoic acid
RA: Retinoic acid
PEA: Palmitoylethanolamide
OT: Olfactory training
MSCs: mesenchymal stem cells
siRNAs: Small interfering RNAs
DRD2: Dopamine D2 receptor
